# *In silico* and in vitro analysis of microRNAs with therapeutic potential in atherosclerosis

**DOI:** 10.1038/s41598-022-24260-z

**Published:** 2022-11-25

**Authors:** Maryam Mahjoubin-Tehran, Seyed Hamid Aghaee-Bakhtiari, Amirhossein Sahebkar, Alexandra E. Butler, Reza Kazemi Oskuee, Amin Jalili

**Affiliations:** 1 Department of Medical Biotechnology and Nanotechnology, Faculty of Medicine, Mashhad University of Medical Sciences, Mashhad, Iran; 2 Bioinformatics Research Group, Mashhad University of Medical Sciences, Mashhad, Iran; 3 Applied Biomedical Research Center, Mashhad University of Medical Sciences, Mashhad, Iran; 4 Biotechnology Research Center, Pharmaceutical Technology Institute, Mashhad University of Medical Sciences, Mashhad, Iran; 5 Department of Biotechnology, School of Pharmacy, Mashhad University of Medical Sciences, Mashhad, Iran; 6 Research Department, Royal College of Surgeons in Ireland, Bahrain, Adliya, Bahrain; 7 Targeted Drug Delivery Research Center, Institute of Pharmaceutical Technology, Mashhad University of Medical Sciences, Mashhad, Iran

**Keywords:** Drug discovery, Cardiology

## Abstract

Atherosclerosis is a chronic inflammatory disease in which aberrant lipid metabolism plays a key role. MicroRNAs (miRNAs), micro-coordinators of gene expression, have been recently proposed as novel clinical biomarkers and potential therapeutic tools for a broad spectrum of diseases. This study aimed to identify miRNAs with therapeutic potential in atherosclerosis. Bioinformatic databases, including experimentally validated and computational prediction tools as well as a novel combination method, were used to identify miRNAs that are able to simultaneously inhibit key genes related to the pathogenesis of atherosclerosis. Further validation of genes and miRNAs was conducted using the STRING online tool, KEGG pathway analysis and DIANA-miRPath. The inhibitory effects of the identified miRNAs in HepG2 and Huh7 cells were verified by real-time PCR. The MTT assay was utilized to evaluate cell cytotoxicity effects of miRNAs. Atherosclerotic drug-targeted genes were selected as key genes. Strong interactions between genes were confirmed using STRING. These genes were shown to be integral to critical pathological processes involved in atherosclerosis. A novel combined method of validated and predicted tools for the identification of effective miRNAs was defined as the combination score (C-Score). Bioinformatic analysis showed that hsa-miR-124-3p and hsa-miR-16-5p possessed the best C-Score (0.68 and 0.62, respectively). KEGG and DIANA-miRPath analysis showed that selected genes and identified miRNAs were involved in atherosclerosis-related pathways. Compared with the controls in both HepG2 and Huh7 cell lines, miR-124 significantly reduced the expression of CETP, PCSK9, MTTP, and APOB, and miR-16 significantly reduced the expression of APOCIII, CETP, HMGCR, PCSK9, MTTP, and APOB, respectively. The cytotoxicity assay showed that miR-124 reduced cell viability, especially after 72 h; however, miR-16 did not show any significant cytotoxicity in either cell line. Our findings indicate that hsa-miR-124 and miR-16 have potential for use as therapeutic candidates in the treatment of atherosclerosis.

## Introduction

Atherosclerosis is known to be a chronic inflammatory disease characterized by lipid accumulation in arteries. Atherosclerosis is largely triggered by the accumulation of lipoproteins containing apolipoprotein B (ApoB), such as LDL and VLDL particles, in blood vessels^1^. According to a recent report, 27.8% of US adults have elevated LDL-C levels (≥ 130 mg/dL)^2^. Advanced atherosclerotic lesions are highly prone to rupture, hemorrhage and thrombus formation. These atherosclerotic complications lead to various diseases such as myocardial infarction, coronary death and stroke^3^. On average, one person dies of cardiovascular disease (CVD) every 36 s in the United States. Based on recent reports, there are about 2396 deaths in the US from CVD each day^2^. Despite advances in developing novel therapies^4–10^, cardiovascular diseases, mainly atherosclerosis, are still the leading causes of death in the world, and these have been predicted to increase in the coming decades^3^. To circumvent the limitations of conventional therapies, there is an obvious need to provide multi-pronged therapeutic approaches that largely focus on the risk factors involved in these diseases^11–13^. Recently, nucleic acid-based therapies have been considerably developed and have revealed promising potential as a therapeutic platform in the treatment of a variety of diseases, some of which have been approved by the US Food and Drug Administration (FDA)^13,14^. Clinical trial-based studies have demonstrated the efficacy of these therapeutics in the treatment of atherosclerosis. Among them, RNA-based therapeutics, specifically small interfering RNAs (siRNAs) and antisense oligonucleotides (ASO) to miRNAs, exhibit great potential as therapeutic candidates in the treatment of diseases such as atherosclerosis^13,15^.

MicroRNAs (miRNAs) are small endogenous noncoding RNAs that act as silencing factors at the post-transcriptional level^16^. The seed region that is located at the 5′ end of the miRNA is an important sequence which binds to the 3′ untranslated regions (UTR) of the target mRNA; consequently, either the mRNA translation is inhibited or the mRNA becomes truncated, unstable and, hence, inactive^17^. Additionally, some studies have shown that miRNAs are able to induce protein truncation with 3′ UTR binding^18^. Recently, evidence has emerged that miRNAs are also able to interact with the promoter and activate gene expression^19^ or alter protein function^20^. Accumulating evidence has revealed that miRNAs can be used as therapeutic, diagnostic, and prognostic tools for an array of disease states^6,11,21–26^.

The overwhelming majority of studies are predicated upon the dogmatic concept that miRNAs regulate the expression of specific target mRNAs by inhibiting mRNA translation or promoting mRNA decay in the RNA-induced silencing complex (RISC). These research efforts have mostly identified and dissected the contributions of multiple regulatory networks of miRNA-target mRNAs to cardiovascular pathogenesis. However, evidence from studies in the past decade has indicated that miRNAs also operate beyond this canonical paradigm, exhibiting non-conventional regulatory functions and cellular localizations that play critical roles in cardiovascular disease^27^.

SiRNAs are another small RNA form that could be utilized in nucleic acid-based therapeutics, and these have recently demonstrated good results for preventive treatment of lipid levels in atherosclerosis. SiRNAs bind perfectly to their targets which are 100% complementary and interfere with transcripts. In contrast to siRNAs, miRNAs binding to target mRNA is imperfect and is done through nucleotides that are located in the seed sequence^11^. This type of binding enables miRNAs to bind and inhibit multiple mRNA targets and, therefore, miRNAs could be viewed as essential epigenetic regulators in the pathogenesis of many diseases. This unique and extraordinary ability of a single miRNA for targeting multiple mRNAs potentially makes them a novel and highly effective therapeutic candidate for the treatment of atherosclerosis^11^.

Numerous clinical trials have established the importance of LDL-C lowering therapy in the primary and secondary prevention of atherosclerosis. Despite reductions in LDL-C with statins, dual lipid-lowering therapy has been shown to be more effective than statin monotherapy in high-risk patients with coronary artery disease, suggesting the attempts to develop new lipid-lowering agents during the recent decades^10,28,29^. Therefore, we aimed to simultaneously reduce LDL-C by inhibition of well-known drug-target genes^30,31^.

**Figure 1:**
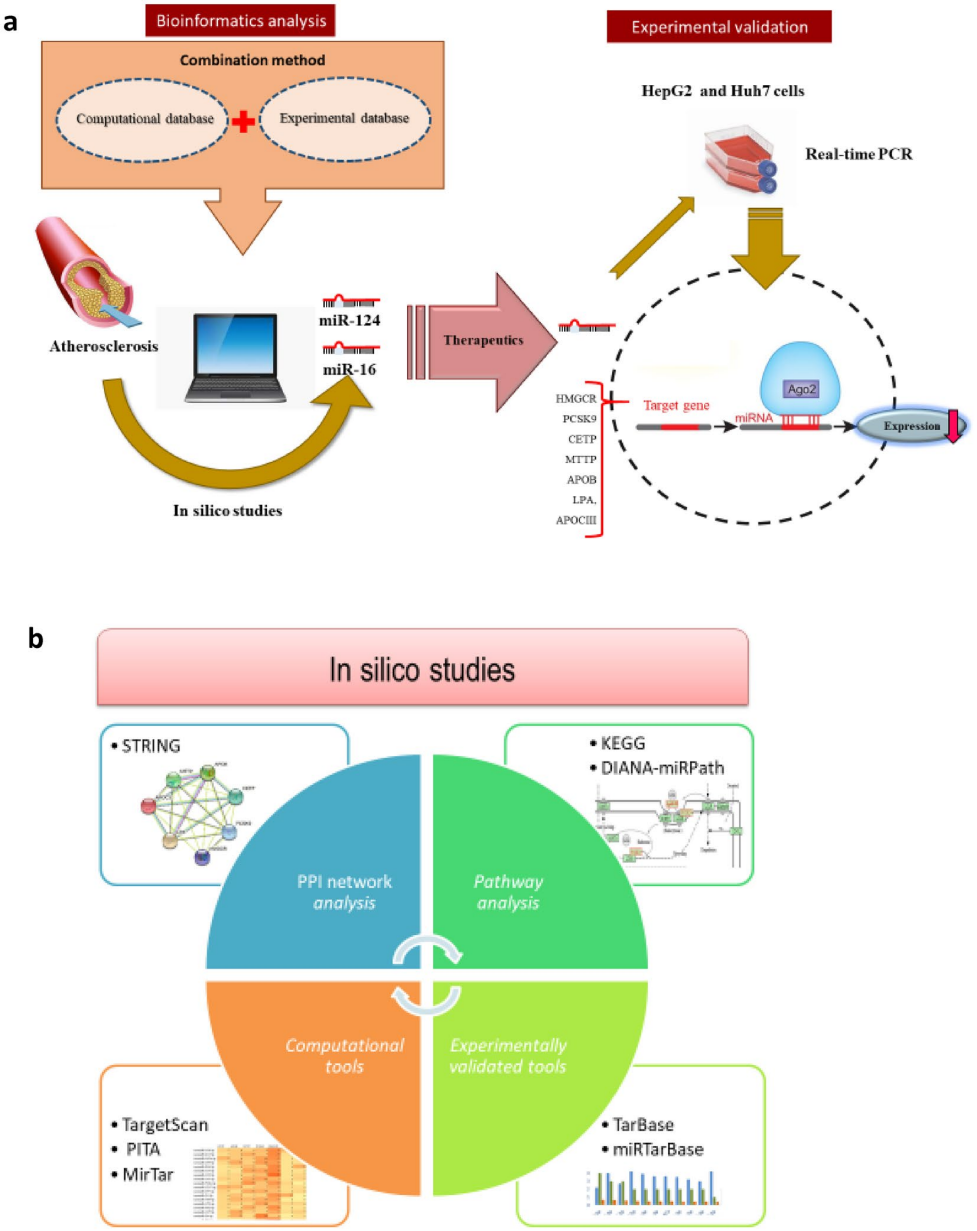
Schematic illustration of the present study. (**a**) Schematic illustration of the study design and results. (**b**) *In silico* analyses used in this study including PPI network analysis, pathway analysis, computational tools, and experimentally validated tools.

Regulation of several targets in a multi-pronged approach using miRNAs in order to improve atherosclerosis could be considered as a promising therapeutic approach^32,33^. To develop effective therapeutics platforms to this end, one needs an efficient approach for the identification and prediction of potentially effective miRNAs in atherosclerosis. Many web-based bioinformatic tools have recently been developed to make these predictions. Two main categories of miRNA prediction tools, computational and experimentally validated tools, are available^16,34^. However, their predictions suffer from poor accuracy and sensitivity as revealed by experimental data. Therefore, to date, no single method has consistently outperformed the others, thus supporting the concept that database content combination is an efficient way to improve predictive relevance^16,35^. Furthermore, another key approach for providing a valuable therapeutics tool for atherosclerosis is selecting effective genes which, if inhibited, could suppress atherosclerosis. Hence, in this study, we selected drug-targeted genes whose efficacy for atherosclerosis treatment had been confirmed in clinical trials (Fig. 1); the protein–protein interaction network of these genes was confirmed using STRING analysis. Moreover, the involvement of selected genes in atherosclerosis was confirmed through KEGG pathway analysis. Thereafter, we identified miRNAs that target these genes based on computational and experimentally validated tools as well as novel combinational methods. The involvement of selected miRNAs in atherosclerosis was confirmed through DIANA-miRPath, and their inhibitory effect on gene expression was verified by real-time PCR.

## Materials and methods

### In silico analysis

#### Drug-target genes as key genes in atherosclerosis pathogenesis

Selection of important genes is the first and crucial step in the identification of effective miRNA in atherosclerosis. The effectiveness of the selected genes should be confirmed; therefore, in the present study we selected drug-target genes whose effectiveness has been confirmed in clinical studies: PCSK9, HMGCR, MTTP, APOCIII, LPA, CETP and APOB.

#### Protein–protein interaction network integration

The STRING (version 11.5) online tool (https://string-db.org/) was used for the identification of protein–protein interactions (PPI) and to explore the relationship between selected genes. It provides direct (physical) interactions and indirect (functional) associations between proteins. We utilized STRING to examine the potential relationships between these candidate genes. The interactions were analyzed with the different available confidence scores and the full STRING network using the medium confidence score was visualized using Cytoscape 3.9.1. The number of node degrees and the PPI enrichment p-value were determined.

#### MiRNA analysis via experimentally validated tools

miRNA analysis by experimentally validated tools usually leads to more accurate results and has less false predictions than computational tools because the information in these tools is based upon experimental studies^36^. We used TarBase v.8^37^ and miRTarBase v.8^16^, two widely comprehensive and up-to-date database validated tools, for the prediction of gene-miRNA interactions. In these databases, we searched each gene separately and summed all the results obtained. Then we listed all miRNAs which could target more than one gene. Theses miRNAs were sorted by validated score (V-score). The V-score was defined for each predicted miRNA as the sum of the number of genes that have been confirmed to be targeted by that miRNA through experimentally validated databases.

### MiRNA analysis via computational tools

Despite the accuracy of validated tools, the number of developed experimentally validated tools is not large. Contrary to empirical tools, computational tools are widely available. Several approaches are used for the prediction of gene-miRNA interactions. The most common method is the utility of seed region sequences as miRNA traits for base pairing between the miRNA and target mRNA. Online computational tools usually are based on the target prediction algorithms, by, for example, checking the thermodynamic stability of the predicted gene-miRNA duplex. To reduce false prediction and improve the strength of the prediction by these algorithms^36^, we used a large number (up to 30) of these computational web-based tools such as TargetScan, PITA, DIANA, MirTar, and miRDB (Supplementary Table 1). Each gene was searched separately, then all obtained results were summed. Next, we created a list of miRNAs that could target more than one gene. Finally, the list of miRNAs was sorted by Predicted score (P-score). The P-score was defined for each predicted miRNA as the sum of the number of computational databases that have confirmed each gene-miRNA interaction. Moreover, further binding characteristics between miRNAs and their target genes were performed using RNAhybrid 2.2 (https://bibiserv.cebitec.uni-bielefeld.de/rnahybrid).

#### Integration of predicted miRNAs

To integrate the results of the two types of tools and achieve the optimal miRNA that target the selected genes, we defined a new score, termed the combined-score (C-score), based on both the computational and experimentally validated tools. The maximum number for a C-Score is 2, achieved when all 30 computational databases verified all 7 selected genes as well as all genes verified by validated databases.

$$ {\mathbf{C}} - {\mathbf{Score}} \, = \frac{{{\varvec{P}} - {\varvec{Score}}}}{{30 \times {\text{Number}}\,{\text{ of}}\,{\text{ target}}\,{\text{ genes}}}} + \frac{V - Score}{{{\text{Number}}\,{\text{ of}}\,{\text{ target}}\,{\text{ genes}}}} $$

#### Pathway analysis for genes and miRNAs

Pathway analysis for target genes was determined by the Kyoto Encyclopedia of Genes and Genomes (KEGG) (https://www.genome.jp/kegg/pathway). KEGG is a publicly accessible knowledgebase, composed of manually curated pathways that cover a wide range of metabolic, genetic, environmental, and cellular processes and human diseases^38–41^.

MiRNA pathway analysis was explored by the online software DIANA-miRPath (version 3.0) (http://www.microrna.gr/miRPathv3). DIANA-miRPath is a web server established for the identification of KEGG pathways corresponding to networks of miRNA targets by superimposing numerous miRNA-target relationships and merging using the meta-analysis algorithm ^42^. This program predicts miRNA targets with high accuracy based on the DIANA-microT-CDS algorithm that considers the evolutionary conservation of miRNA-binding sites.

### In vitro studies

#### Cell culture

Human hepatocyte cell lines (HepG2 and Huh7) were used to investigate the effect of miRNAs on the expression of target genes^17,18^. We obtained cell lines from the Cell Bank of Pasteur Institute of Iran (Tehran). Cells were maintained in RPMI-1640 supplemented with 10% FBS and in a humidified incubator at 37 °C and 5% CO2.

#### Plasmid construct and extraction

The pLenti-III-miR124-GFP and pLenti-III-miR16-GFP expression vector construct and pLenti-III-GFP empty vector (control vector) were purchased from the Stem Cell Technology Research Center vector bank (STRC, Tehran, Iran). The *E. coli* *Stbl4* strain harboring the vectors was cultured in LB broth medium with 25 µg/mL kanamycin. The plasmid DNA was purified from overnight cultures of *E. coli* with the Qiagen EndoFree Plasmid Maxi Kit (Qiagen, Hilden, Germany) according to the manufacturer’s guidelines.

#### Transfection

HepG2 and Huh7 were seeded in 24-well culture plates with 3 × 10^4^ cells/well and 1.2 × 10^5^ cells/well, and incubated at 37 °C with 5% CO2. pLenti-III-miR124-GFP and pLenti-III-miR16-GFP expression vector construct and pLenti-III-GFP empty vector (as the control in every experiment) was transfected into the cells with the transfection reagent PolyFect (Qiagen, Hilden, Germany) according to manufacturer’s guidelines. Transfection efficiency after 24, 48, and 72 h was determined by fluorescent microscopy. 48 h after transfection (the timepoint with the strongest GFP signal), the cells were harvested for RNA Extraction and Quantitative RT-PCR.

#### RNA extraction and quantitative RT-PCR

Cell pellets were collected using 0.25% EDTA-trypsin prior to RNA extraction. mRNA and miRNA extraction were performed using a modified RNX-Plus RNA extraction kit (SinaClon, Iran)^43,44^. In short, 2–3 × 10^6^ cells from the HepG2 and Huh7 cell lines were suspended in 1 ml RNX-Plus. After 5 min incubation at room temperature, 250 μl chloroform (Merck, Germany) was added to each tube and the tubes were shaken vigorously. The tubes were then centrifuged by MIKRO 200 R (Hettich, Germany) for 25 min at 12,000 g and 4 °C. The upper phase was transferred to a new tube and an equal volume of 99% ethanol (Merck, Germany) was added. The tubes were kept at − 20 °C overnight and subsequently centrifuged for 45 min at 12,000 g and 4 °C. The supernatant was then removed and 1 ml of 70% ethanol was added. The tubes were centrifuged for 20 min at 12,000 g and 4 °C. The supernatant was discarded and the RNA pellets were dried at room temperature. The quality and purity of the RNA was analyzed by BioPhotometer (Eppendorf, Germany). The RNA samples were stored at − 80 °C until further use. Total RNA was extracted and complementary DNA (cDNA) synthesis was carried out with reverse transcriptase. Oligo dT primers and designed stem-loop primers (www.mirbase.org) were used for reverse transcription of genes and miRNAs, respectively (Table 1). Real-time PCR was performed with SYBR Premix Dimer EraserTM (TaKaRa) and analyzed using the Roche LightCycler® 96 System. Forward and reverse primers were used for real-time PCR and designed using AlleleID, Gene Runner, and OLIGO primer analysis Software. All reactions were performed in triplicate. The relative expression levels of mRNA and miRNA were calculated by the 2^−ΔΔCT^ method^45^ and normalized to GAPDH and SNORD 47 (U47) reference genes, respectively.

**Table 1:** The sequences of primers used for cDNA synthesis and qRT-PCR reaction.

Gene name	Primer sequences (5′–3′)
hsa-miR-124-3p	F primer: CTAAGGCACGAGGTGAA
	R primer: GAGCAGGGTCCGAGGT
	Stem-loop primer: GTCGTATCCAGAGCAGGGTCCGAGGTATTCGCACTGGATACGACTTGGCA
hsa-miR-16-5p	F primer: GCCTAGCAGCACGTAAATA
	R primer: GAGCAGGGTCCGAGGT
	Stem-loop primer: GTCGTATCCAGAGCAGGGTCCGAGGTATTCGCACTGGATACGACCGCCAA
SNORD 47	F primer: CGCTTCGGCAGCACATATACTA
	R primer: GGAACGCTTCACGAATTTGC
	Stem-loop primer: GGAACGCTTCACGAATTTGC
PCSK9	F primer: GAACCTGGAGCGGATTACC
	R primer: TGCTGGCCTGTCTGTGGA
ApoCIII	F primer: GCTCCAGGAACAGAGGTGC
	R primer: CGTGCTTCATGTAACCCTGC
ApoB	F primer: CGGTCAACAACTATCATAAG
	R primer: ACCCGCAGAATCAAATAGG
MTTP	F primer: AATAGATCATTCTCAGGAAC
	R primer: GTGATGTCAGTGGTTATTAC
LPA	F primer: TCCAGATGCCGATACAGG
	R primer: CATACAGTCTTGTTCAGAAGG
HMGCR	F primer: CAGTTGTCATTCACTTCTTAG
	R primer: CTTCATCCTGTGAGTTGG
CETP	F primer: CCAGATCAGCCACTTGTCC
	R primer: ATCAATACCCAGCCACCAG
GAPDH	F primer: CCTCAAGATCATCAGCAATG
	R primer: CATCACGCCACAGTTTCC
Oligo dT (18)	TTTTTTTTTTTTTTTTTT

F and R primers used for each miRNA or gene in qRT-PCR and specific stem-loop primers and oligo dT primers used in cDNA synthesis for miRNAs and genes, respectively.

*F* forward primer, *R* reverse primer.

#### Cell viability assay

HepG2 and Huh7 cells were seeded in 96-well culture plates with 1.5 × 10^4^ cells/well and 4 × 10^3^ cells/well, and incubated at 37 °C with 5% CO_2_. The medium was replaced with a fresh serum-free medium after incubating for 24 h, and each group of cells was transfected with miR-124, miR-16 and empty vector and the plates were then incubated for the next 24, 48, and 72 h. Untreated cells were used as controls. Afterwards, 10 μl MTT solution (5 mg/ml) was added to the cells in each well and incubated for 3 h. Formazan crystals were then dissolved in 100 μl dimethyl sulfoxide. After rocking for 10 min, the absorbance value of each well was measured with a microplate reader (BioTek, Richmond, USA) at 570 nm and 630 nm as the reference wavelength.

#### Statistical analysis

Data is presented as mean ± standard deviation (SD) of at least three independent experiments. IBM SPSS Statistics V22.0 software was used for statistical analysis. Statistical significance between groups was assessed using the one-way analysis of variance (ANOVA) followed by Tukey’s post hoc analysis. In each experiment, the results were normalized using treatment with empty vectors and their significance was determined. In addition, the Kolmogorov–Smirnov method was used for normality test analysis using SPSS. Differences were considered significant at the *p < 0.05 and **p < 0.01 level.

## Results

### Drug-target genes

To select the effective genes which, if inhibited, could suppress atherosclerosis, in this study we selected drug-targeted genes whose efficacy for atherosclerosis treatment had been previously confirmed in clinical trials. β-hydroxy-β methylglutaryl Coenzyme A Reductase (HMGCR), proprotein convertase subtilisin–kexin type 9 (PCSK9), cholesteryl ester transfer protein (CETP), microsomal triglyceride transfer protein (MTTP), apolipoprotein B (APOB), lipoprotein A (LPA), and apolipoprotein C (APOCIII) were candidate genes in this study. The effectiveness of these genes in atherosclerosis has been confirmed in different clinical trials (Table 2).

**Table 2:** Selective key genes based upon clinical research.

Symbol	Gene name	Drug	Targeting approach	NCT number
HMGCR	β-hydroxy-β methylglutaryl Coenzyme A Reductase	Statin	HMGCR inhibitor	NCT00965185 NCT03354156 NCT00560170
PCSK9	proprotein convertase subtilisin–kexin type 9	Inclisiran	GalNAc-siRNA that inhibits translation of PCSK9	NCT02597127 NCT03399370 NCT03397121
CETP	Cholesteryl ester transfer protein	Anacetrapib	CETP inhibitor	NCT02931188 NCT01252953
MTTP	Microsomal triglyceride transfer protein	Lomitapide	MTTP inhibitor	NCT02173158 NCT02399852 NCT00943306
APOB	Apolipoprotein B	Mipomersen	ASO that inhibits translation of APOB	NCT01475825 NCT01598948 NCT00477594
LPA	Lipoprotein (a)	IONIS-APO(a)Rx	GalNAc-ASO that inhibits translation of LPA	NCT02414594 NCT02160899
APOCIII	Apolipoprotein C	Volanesorsen	ASO that inhibits translation of POCIII	NCT02300233 NCT02658175 NCT01529424

The effectiveness of these genes was verified in clinical trials. Key trials, with NCT number, are provided in the table.

### PPI analysis

**Figure 2:**
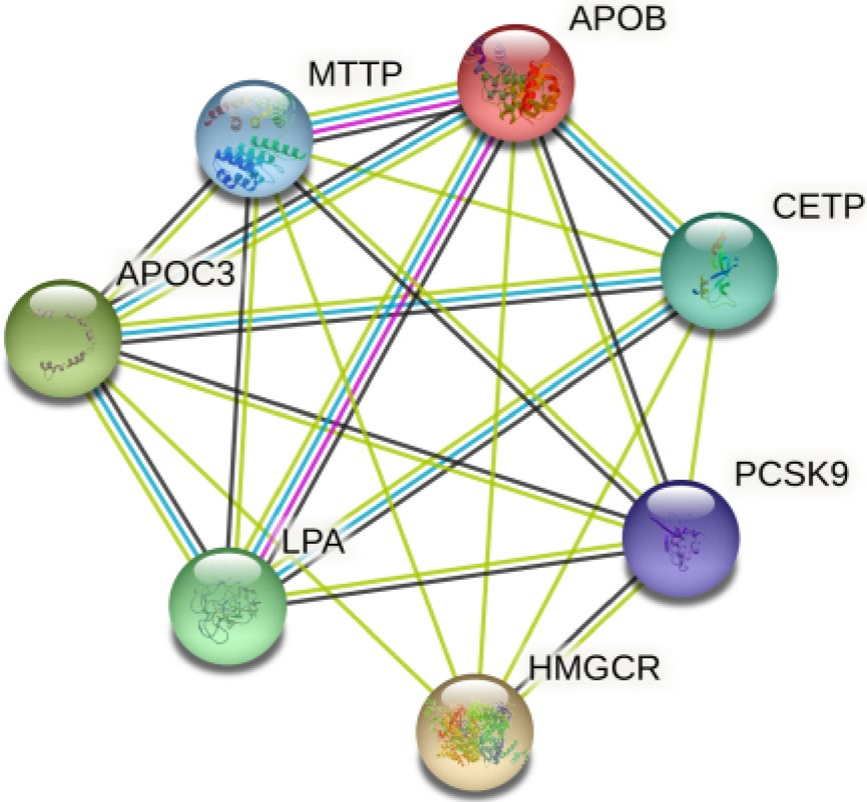
The PPI network for the selected genes using the medium confidence score. The circles represent the proteins encoded by the corresponding genes; edges represent the interactions between the proteins and the predicted functional associations. PPI enrichment *p* value < 1.0e−16.

**Figure 3:**
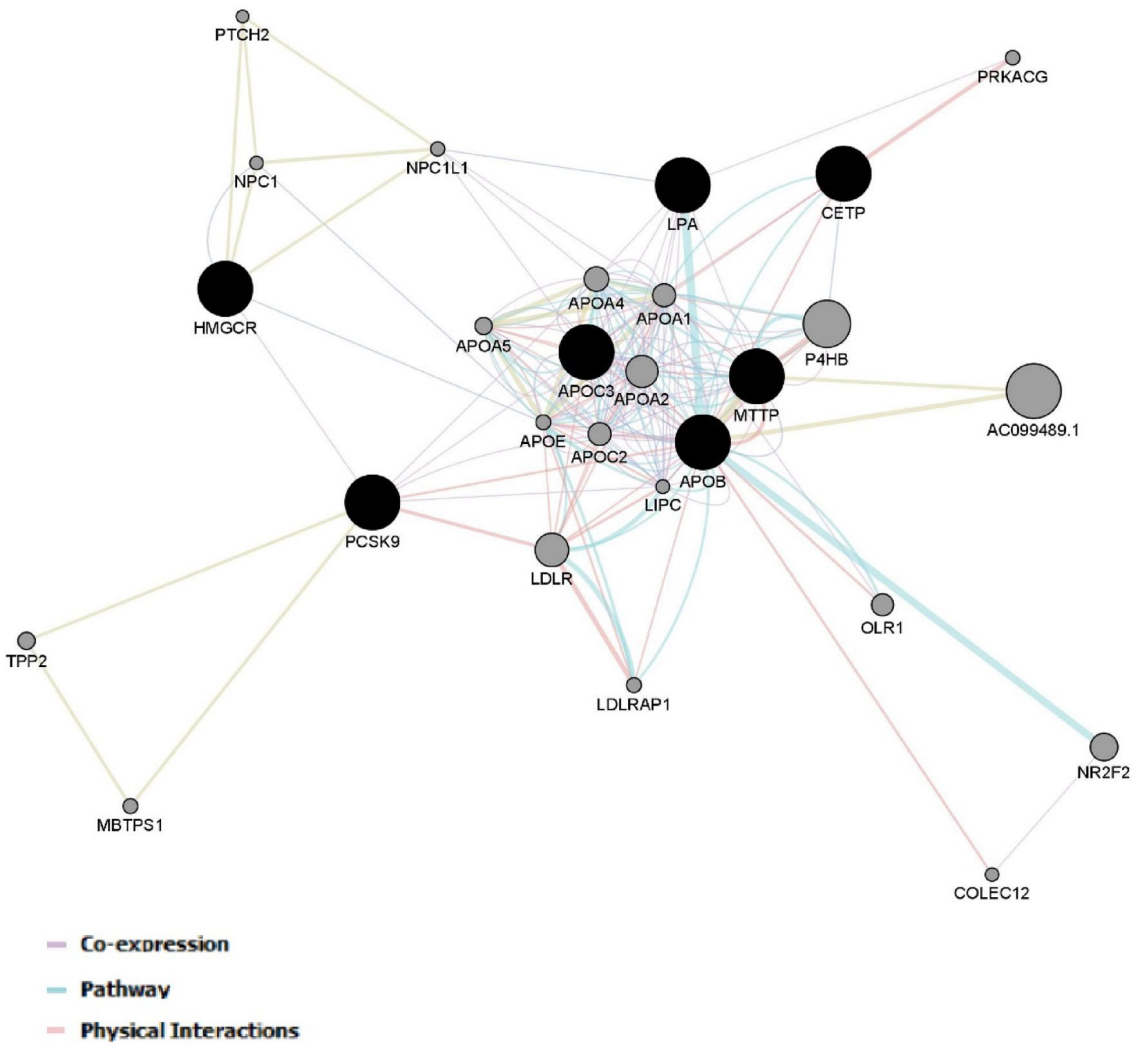
A co-expression network for the selected genes was constructed using the GeneMANIA plugin of Cytoscape^47^. A set of selected genes were provided as a query (black nodes) and a number of additional genes were predicted to be related (gray nodes).

The STRING online analysis tool was used to show the PPI network between the candidate genes. The interactions among the proteins are depicted by confidence scores that range between 0 and 1. The highest confidence score lies between 0.9 and 1, high confidence score between 0.7 and 0.9, medium confidence score between 0.4 and 0.7 and low confidence score is less than 0.4. In the network, each gene is designated as a node and the interactions between the nodes are designated as edges^46^. The results of the analysis with highest, high, medium and low confidence scores are 7, 8, < 1.11e−16; 7, 14, < 1.0e−16; 7, 20, < 1.0e−16 (nodes, edges, and *p* value, respectively). The PPI network using the medium confidence score is shown in Fig. 2. Using this confidence score, interactions between MTTP, APOB, and LPA were experimentally determined. Interactions between CETP, APOC3, MTTP, APOB and LPA were from curated databases. Other interactions were from Predicted Interactions. These genes are mainly involved in the process of LDL remodeling, VLDL assembly, chylomicron assembly and HDL remodeling. All these pathological processes play critical roles in atherosclerosis. Moreover, a co-expression network for the selected genes was constructed by mapping the selected genes into an extensive database of functional-interaction datasets in the GeneMANIA plugin of Cytoscape (Fig. 3).

**Figure 4:**
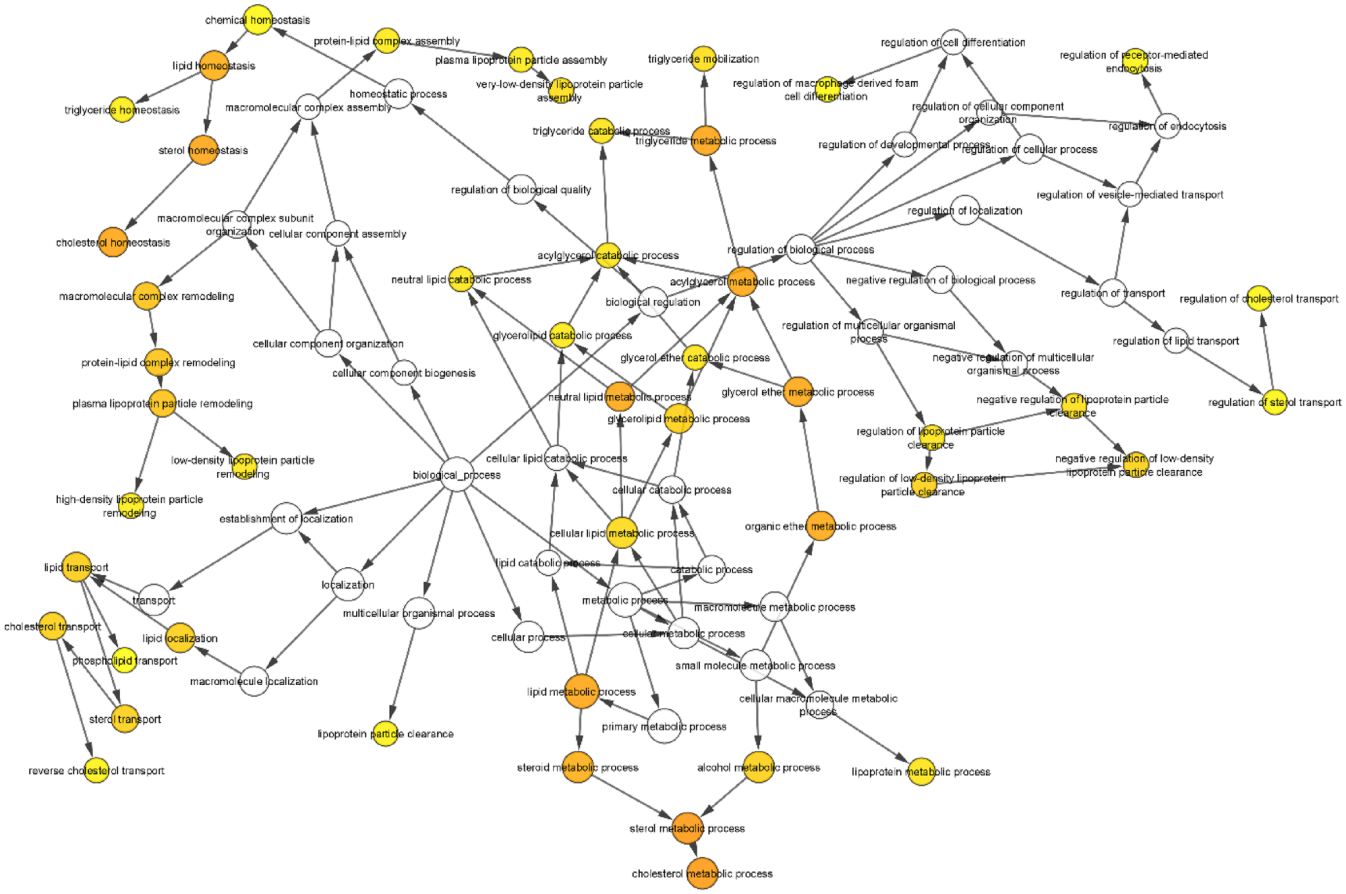
Biological process analysis was used to confirm the critical genes in the pathophysiology of atherosclerosis using the CytoCluster Plugin in Cytoscape^48^.

To further confirm the critical genes in the pathophysiology of atherosclerosis, the CytoCluster Plugin in Cytoscape^48^ was used (Fig. 4). In addition, biological process analysis for atherosclerosis pathogenesis was performed using the CytoCluster Plugin in Cytoscape (Table 3).

**Table 3:** Biological process analysis for atherosclerosis pathogenesis.

Atherosclerosis related process	*p* value	Cluster frequency (%)	Genes
Cholesterol metabolic process	1.94E−10	7/7 100.0	CETP MTTP APOC3 PCSK9 HMGCR APOB LPA
Sterol metabolic process	3.13E−10	5/7 71.4	CETP APOC3 PCSK9 HMGCR APOB
Triglyceride metabolic process	2.23E−09	4/7 57.1	CETP APOC3 PCSK9 APOB
Lipid metabolic process	2.23E−09	7/7 100.0	CETP MTTP APOC3 PCSK9 HMGCR APOB LPA
Cholesterol homeostasis	2.71E−09	4/7 57.1	CETP APOC3 PCSK9 APOB
Sterol homeostasis	2.71E−09	4/7 57.1	CETP APOC3 PCSK9 APOB
Acylglycerol metabolic process	3.88E−09	4/7 57.1	CETP APOC3 PCSK9 APOB
Neutral lipid metabolic process	4.22E−09	4/7 57.1	CETP APOC3 PCSK9 APOB
Glycerol ether metabolic process	4.97E−09	4/7 57.1	CETP APOC3 PCSK9 APOB
Lipid homeostasis	1.11E−08	4/7 57.1	CETP APOC3 PCSK9 APOB
Steroid metabolic process	1.27E−08	5/7 71.4	CETP APOC3 PCSK9 HMGCR APOB
Lipid transport	2.92E−07	4/7 57.1	CETP APOC3 APOB LPA
Lipid localization	4.07E−07	4/7 57.1	CETP APOC3 APOB LPA

### MiRNA identification via experimentally validated tools

The top miRNAs which could target selected genes identified by experimentally validated databases (TarBase and miRTarBase) are shown in Table 4. The V-Score is defined as the number of genes that are targeted by the miRNA. The results showed that miR-124 had the highest V-score, targeting CETP, APOB, MTTP and PCSK9. MiR-16 and miR-191 have an equivalent V-Score, targeting three genes (PCSK9, HMGCR, and APOB and PCSK9, APOB, and APOC3, respectively).

**Table 4:** Important miRNAs in atherosclerosis identified by experimentally validated databases.

microRNA	CETP	APOB	MTTP	PCSK9	HMGCR	LPA	APOC3	V-Score
hsa-miR-124-3p	**✓**	✓	✓	✓	–	–	–	4
hsa-miR-16-5p	–	✓	–	✓	✓	–	–	3
hsa-miR-191-5p	–	✓	–	✓		–	✓	3
hsa-miR-335-5p	–	–	–	✓	✓	–	–	2
hsa-miR-29c-3p	–	–	–	✓	✓	–	–	2
hsa-miR-27a-3p	–		–	✓	✓	–	–	2
hsa-miR-147a	–	–	–	✓	–	✓	–	2
hsa-let-7b-5p	–	✓	–	✓	–	–	–	2
hsa-miR-15b-5p	–	–	–	✓	✓	–	–	2
hsa-miR-1915-3p	–	–	–	✓	✓	–	–	2

MiRNA-gene interaction that was confirmed by the validated databases is noted by a check mark in the relevant cells of the table.

### MiRNA identification via computational tools

The results of identification of effective miRNA in atherosclerosis using the aforementioned computational databases are provided in Table 5 (additional results are provided in Supplementary Table 2). In addition, further investigations of binding characteristics between miRNAs and their target genes were performed using RNAhybrid 2.2^49^ (Supplementary Tables 3 and 4).

**Table 5:** Important miRNAs in atherosclerosis identified by computational databases.

miRNAs	CETP	APOB	MTTP	PCSK9	HMGCR	LPA	APOC3	P-Score
hsa-miR-338-3p	5	6	5	8	16	9	8	57
hsa-miR-211-5p	3	6	10	7	16	3	10	55
hsa-miR-365a-3p	9	6	6	12	15	2	2	52
hsa-miR-149-5p	4	11	5	13	13	2	3	51
hsa-miR-204-5p	2	5	8	7	16	2	10	50
hsa-miR-335-5p	2	2	12	12	16	2	2	48
hsa-miR-139-5p	3	4	6	8	20	2	4	47
hsa-miR-143-3p	5	4	10	12	9	2	5	47
hsa-miR-548c-3p	2	5	14	7	14	4		46
hsa-miR-1237-3p	9	9	4	7	10	3	3	45

The number of databases that confirmed each miRNA-gene interaction is provided in each cell. The sum of the number of databases for each miRNA is provided as a P-Score in the final column.

In Table 5, the gene scores represent the number of databases which confirmed each miRNA target for each gene. The first ten results were sorted based on the P-Scores, which are the sum of gene scores for each miRNA. miR-338, miR-211 and miR-365a achieved the highest scores.

### Integration of predicted miRNAs

**Figure 5:**
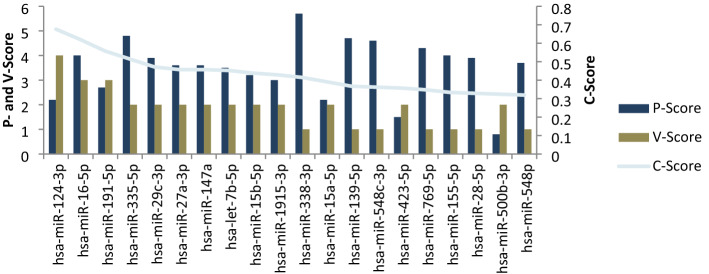
Identified important miRNAs in atherosclerosis based on their P-Score, V-Score and C-Score. The P-Score and the V-Score (column charts on the left axis) are obtained from computational and experimentally validated tools, respectively. The C-Score (line chart on the right axis) is a product of combining the results of both types of databases, which identifies the most important miRNAs.

To identify the most effective miRNAs based on both validated and computational databases, we calculated the C-Score for each miRNA. The C-Score is a combination score that takes into account both previous tools. The miRNAs were sorted based upon their C-Scores. As is shown in Fig. 5, the C-Score is a combination of the P-Score and the V-Score.

### Pathway analysis

**Figure 6:**
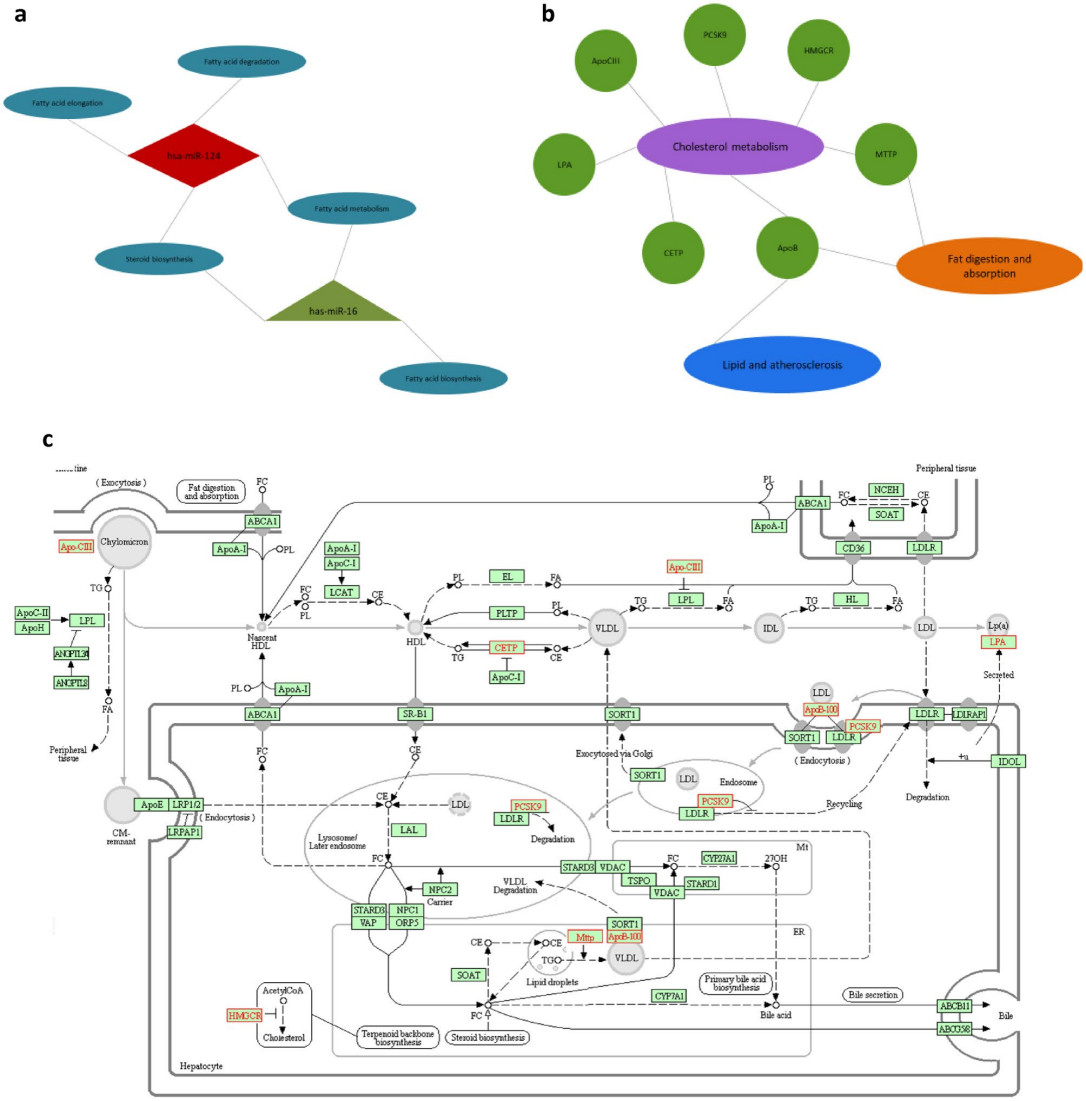
The molecular regulatory interaction network. (**a**) Atherosclerosis-related pathways investigated for miR-124 and miR-16 by DIANA miRPath^42^, (**b**) Pathways involved in atherosclerosis pathogenesis determined by KEGG pathway analysis (https://www.kegg.jp/kegg/kegg1.html)^39–41^. Selected genes have important roles in these pathways. (**c**) The enriched Kyoto Encyclopedia of pathogenesis pathways in atherosclerosis. Red rectangles represent the selected genes (CETP, ApoB, ApoCIII, MTTP, PCSK9, LPA, and HMGCR).

As shown in Fig. 6-a, the DIANA miRPath revealed four and three atherosclerosis-related pathways in which miR-124 and miR-16 have effective roles, respectively. Assessment using the KEGG pathway analysis showed that all selected genes target cholesterol metabolism, a critical pathway in atherosclerosis development (Fig. 6).

### Over expression of miR-124 and miR-16

**Figure 7:**
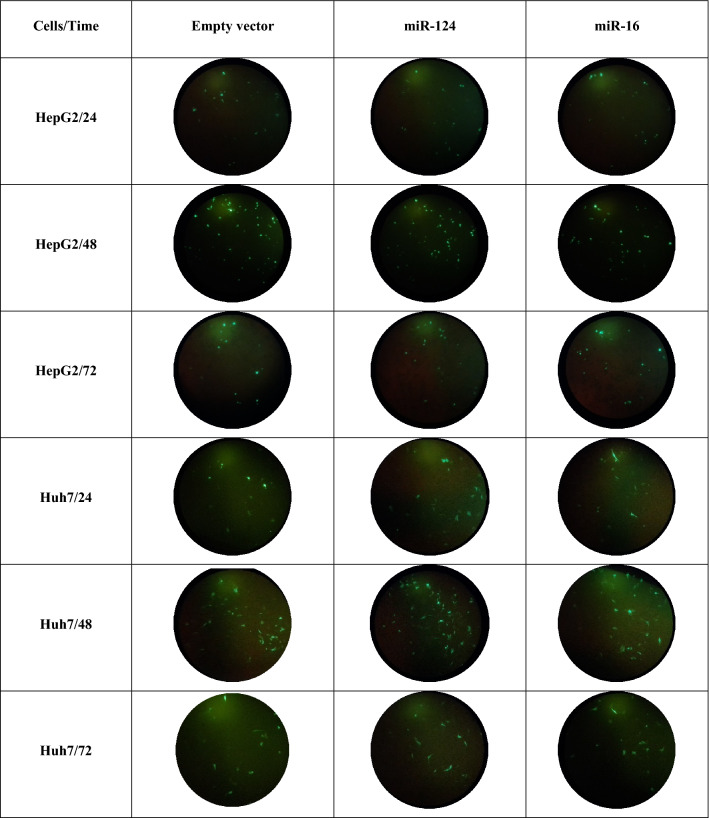
GFP expression of plasmids, including empty vector, for miR-124 and miR-16 at 24, 48 and 72 h post-transfection evaluated by fluorescence microscopy (used as a reporter for miRNA expression in HepG2 cells). At 48 h post-transfection, the optimum expression was found.

**Figure 8:**
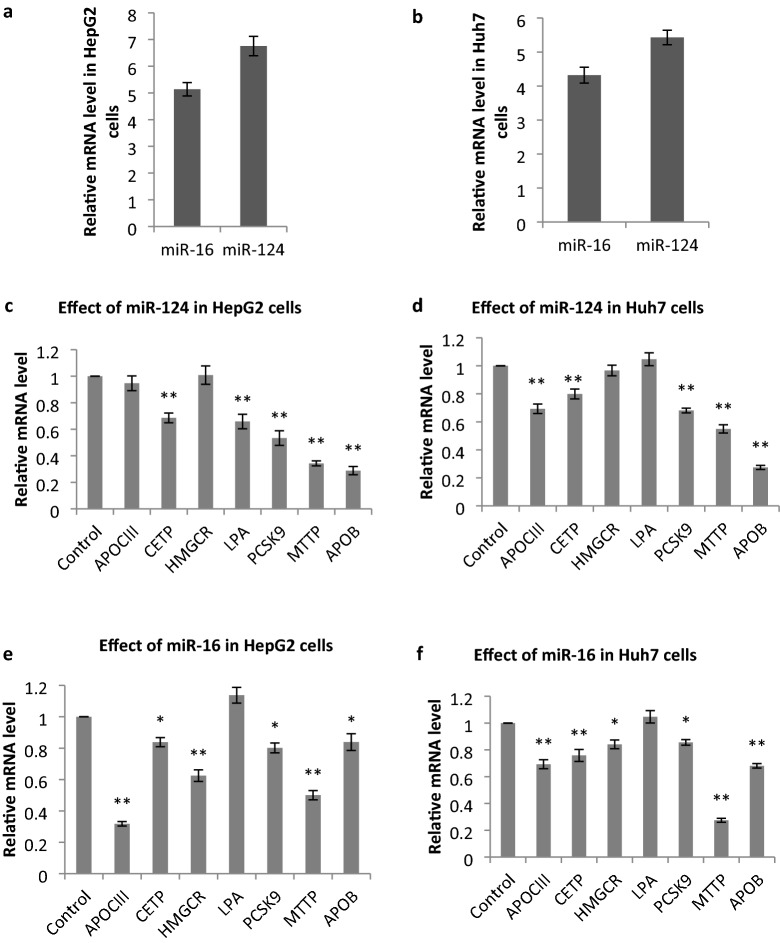
In vitro effects of identified miRNAs on gene expression at 48 h post-transfection. Overexpression of miR-124 (**a**) and miR-16 (**b**) in HepG2 and Huh7 cell lines relative to the control. (**c**) Effect of miR-124 on gene expression in HepG2 cells. (**d**) Effect of miR-124 on gene expression in Huh7 cells. (**e**) Effect of miR-16 on gene expression in HepG2 cells. (**f**). Effect of miR-16 on gene expression in Huh7 cells. Data is presented as mean ± S.D. of at least three independent experiments. Statistical significance between groups was assessed using the one-way ANOVA followed by Tukey’s post hoc analysis. Statistical significance was evaluated relative to the control vector. *p < 0.05 and **p < 0.01.

Transfection efficiency was determined by analyzing the GFP-expressing plasmid. To visualize cells expressing GFP, the plates were observed by fluorescent microscopy. The transfection of plasmids encoding miR-124-3p and miR-16-5p or empty vectors was performed in HepG2 and Huh7 cell lines to verify the effects of these miRNAs. Monitoring of GFP signal at 24, 48, and 72 h post-transfection showed that the optimal expression occurred at 48 h (Fig. 7). To validate whether miR-124 and miR-16 were upregulated in cells, the levels of miRNAs were determined by Real-time PCR 48 h after transfection. We found a 6.75 and 5.43-fold increase in miR-124 expression (*p* < 0.01) and 5.14 and 4.3 (p < 0.01) fold increase in miR-16 expression in HepG2 and Huh7 cells, respectively (Fig. 8a,b).

### Effect of miR-124 on the expression of target genes

To validate the in vitro effect of miR-124 on the expression of the target genes, APOCIII, CETP, HMGCR, LPA, PCSK9, MTTP, APOB, after transfection of miR-124 or empty control vector in HepG2 and Huh7 cell lines, the expression level of target genes was evaluated by q-PCR. Results showed that miR-124 significantly reduced the expression of APOCIII (0.69 ± 0.03; *p* < 0.01) only in Huh7 cells, LPA (0.66 ± 0.05; *p* < 0.01) only in HepG2 cells; miR-124 reduced the expression of the other genes in both cell lines: CETP (0.68 ± 0.04; *p* < 0.01), PCSK9 (0.53 ± 0.05; *p* < 0.01), MTTP (0.34 ± 0.02; *p* < 0.01), and APOB (0.29 ± 0.03; *p* < 0.01) in HepG2 and CETP (0.80 ± 0.04; *p* < 0.01), PCSK9 (0.68 ± 0.02; *p* < 0.01), MTTP (0.55 ± 0.03; *p* < 0.01), and APOB (0.27 ± 0.01; *p* < 0.01) in Huh7 cells, fold changes relative to control ± SD; *p* value, respectively (Fig. 8c, d).

## Effects of miR-16 on the expression of target genes

To analyze the in vitro effect of miR-16, the expression level of target genes was evaluated by q-PCR.

Results revealed that miR-16 significantly reduced the expression of APOCIII (0.32 ± 0.01; *p* < 0.01, 0.69 ± 0.03; *p* < 0.01), CETP (0.84 ± 0.03; *p* < 0.05, 0.76 ± 0.04; *p* < 0.01), HMGCR (0.62 ± 0.04; *p* < 0.01, 0.84 ± 0.03; *p* < 0.05), PCSK9 (0.80 ± 0.03; *p* < 0.05, 0.85 ± 0.02; *p* < 0.05), MTTP (0.50 ± 0.03; *p* < 0.01, 0.27 ± 0.01; *p* < 0.01), and APOB (0.84 ± 0.05; *p* < 0.05, 0.68 ± 0.02; *p* < 0.01), fold changes relative to control ± SD; p-value in HepG2 and Huh7 cells, respectively (Fig. 8e, f).

### Effect of miR-124 and miR-16 on HepG2 and Huh7 cell viability

**Figure 9:**
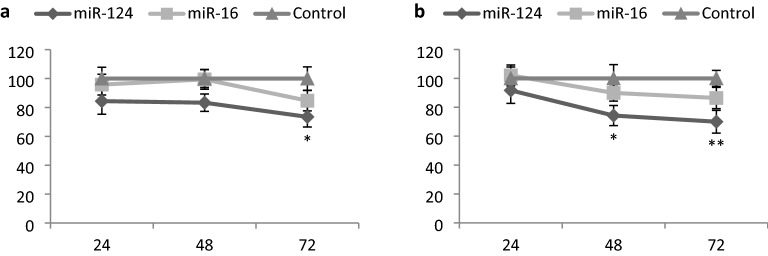
Effect of miR-124 and miR-16 on cell viability of HepG2 cells (**a**) and Huh7 cells (**b**) as determined by the MTT assay. Significance was evaluated compared to the control vector. *p < 0.05 and **p < 0.01.

To evaluate the cell toxicity of the miRNAs, HepG2 and Huh7 cells were transfected with miR-124 and miR-16. The effects of the miRNAs on cell viability were determined by MTT assay compared with the control vector-transfected cells at 24, 48 and 72 h post-transfection (Fig. 9). A trend was seen towards a reduction in cell viability in HepG2 and Huh7 cells by miR-16 overexpression. However, this trend did not reach statistical significance (*p* > 0.05). Overexpression of miR-124 significantly reduced viability in HepG2 only after 72 h and Huh7 after 48 and 72 h.

## Discussion

Despite considerable advances in treatment approaches, atherosclerosis still remains among the leading causes of vascular disease worldwide, encompassing ischemic heart disease, ischemic cerebrovascular disease and peripheral arterial disease^50^. Regulation of several molecular targets in a multi-pronged approach to improve disease control is the aim of modern pharmacology^11^. This approach provides a platform to increase the efficiency of therapeutic tools by targeting various pathways that are involved in the pathogenesis of disorders such as atherosclerosis^32,33^.

MiRNA-based drugs, possessing the ability to target several genes simultaneously, can be potentially considered as a powerful and effective therapeutic tool since they can regulate the expression of multiple genes involved in a disease^51,52^. However, the interactions of miRNA with mRNA is incompletely understood and miRNAs have the potential to regulate other pathways as off-target effects^53^. Therefore, in this study, we aimed to use a comprehensive and precise bioinformatic identification methodology to limit the adverse off-target effects of miRNAs.

Recently, numerous prediction databases and programs have been developed for the identification of miRNA-gene interactions. Mukushkina et al. predicted miRNA interactions with candidate atherosclerosis genes using the MirTarget program^54^. Soh et al.^55^ selected MTTP and Apo B as selected genes and identified microRNA-30c as the targeting miRNA using TargetScan.

Indeed, experimentally validated databases would have a higher predictive value, but due to the lack of sufficient empirical data, this kind of tool has not, to date, been widely developed. As a consequence, computational prediction tools are harnessed to increase the predictability of miRNA-mRNA interactions. Use of both validated and predicted tools helps not only to identify the most effective miRNAs but also to reduce the probability of off-targeting. In this study, we have proposed a combination method to identify the effective miRNAs for the treatment of atherosclerosis based on both parameters.

Effective miRNAs were identified based on targeting seven drug-based genes, namely HMGCR, PCSK9, CETP, MTTP, APOB, LPA and APOCIII, each of which has previously been identified in clinical trials (Table 2). These genes were confirmed to have a strong PPI network as identified by STRING analysis. In this study, hsa-miR-124-3p and hsa-miR-16-5p were identified as effective therapeutic miRNAs for the treatment of atherosclerosis. Assessing miR-124 and miR-16 in vitro verified the results of the bioinformatic analysis.

**Figure 10:**
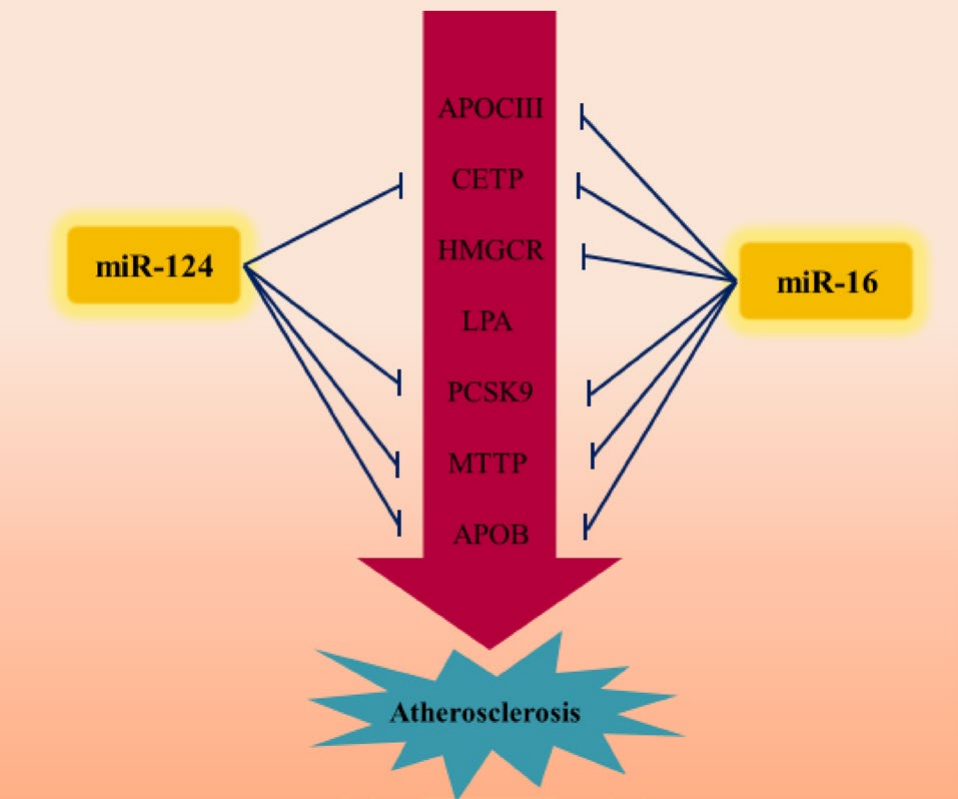
MiR-124 and miR-16 can inhibit the expression of key genes which lead to atherosclerosis.

Through experimental studies, we confirmed that miR-124 could significantly downregulate the expression of CETP, PCSK9, MTTP, and APOB, and miR-16 could significantly downregulate the expression of APOCIII, PCSK9, CETP, HMGCR, MTTP, and APOB (Fig. 10). Although a significant concordance between *in silico* and in vitro results was observed, there are some notable discrepancies between them. For example, validated databases did not identify that miR-124 regulates LPA and that miR-16 regulates MTTP, CETP and APOC3 as was found here experimentally. This deficiency is likely due to the limited experimental studies that are available in these databases. Interestingly, these interactions were identified by predicted databases and this indicates the importance of using our combinational method instead of only one type of database. Furthermore, in some cases, predicted databases showed that miR-124 and miR-16 could target HMGCR and LPA (by low score), respectively; however, our in vitro results showed that these miRNAs did not affect the expression of these genes. It can thus be concluded that the predicted databases sometimes present false positive results. Therefore, in order to increase the accuracy of the results, more predicted databases should be applied and the results of the validated databases could also be used to verify the results of the predicted databases.

MiR-124, as an important modulator of cell proliferation and differentiation, attenuated human aortic vascular smooth muscle proliferation^56^. Choe et al. showed that miR-124 could inhibit neointimal formation by decreasing VSMC proliferation^57^. Liang et al. introduced miR-124 as a promising treatment for atherosclerosis. They demonstrated that miR-124 overexpression inhibited macrophage apoptosis through increasing the expression of anti-inflammatory cytokines and reducing the expression of pro-inflammatory cytokines^58^.

Expression of miR-16 was downregulated in animal models of atherosclerosis and in macrophage-derived foam cells. Liang et al. also found that miR-16 suppressed the activation of inflammatory macrophages in atherosclerosis. Additionally, they showed that miR-16 reduced the secretion and mRNA expression of proinflammatory factors, whereas it enhanced the secretion and mRNA expression of the anti-inflammatory factor through downregulation of PDCD4 and activation of p38^59^. Wang et al. demonstrated that miR-16 overexpression inhibited foam cell formation by exerting anti-inflammatory effects. They found that miR-16 in Apoe-/- mice reduced the formation of atherosclerotic plaques and suppressed the accumulation of proinflammatory factors in the plasma and tissues but promoted the secretion of anti-inflammatory factors. Moreover, they found that miR-16 was downregulated in the plasma and peripheral blood mononuclear cells of CAD patients^60^.

Cell viability assays showed that miR-124 but not miR-16 had low cytotoxicity, which may be due to the anticancer effect of miR-124^61^. To overcome this obstacle, one approach is to use low doses of combined miRNAs that synergistically regulate the expression of the same target genes. Such a gene regulation approach would offer a complementary effect^62^.

Overall, our study showed the effect of miR-124 and miR-16 on inhibiting the expression of key genes involved in atherosclerosis. Moreover, our additional database studies showed that miR-124 and miR-16 could also target Interleukin-1 beta (IL-1β) as one of the potential avenues for targeting inflammation in atherosclerosis. Notably, miR-16 not only inhibited more genes but also did not show any cytotoxicity in both tested hepatic cell lines. Further studies including *vivo* investigations are needed to assess and confirm the therapeutic and adverse effects of miR-124 and miR-16. The main advantage of these miRNAs over other therapeutic oligonucleotides for the inhibition of gene expression such as siRNAs, such as inclisiran, or ASOs, such as mipomersen, is that miRNAs could simultaneously silence the expression of multiple genes.

Future studies could reveal the inhibitory effects of these miRNAs on protein levels of the target genes as well as lipoprotein production and plaque formation in atherosclerotic animal models. In vivo, delivering miRNAs to target sites such as the liver could minimize toxicity and side effects as well as enhance the efficacy of treatment^63^.

## Supplementary Information

Supplementary Information.

## Data Availability

The data are available from the corresponding author upon a reasonable request.
