# Supplementary material for: In silico and in vitro analysis of microRNAs with therapeutic potential in atherosclerosis
Source: Sci Rep. 2022 Nov 25;12:20334. doi: 10.1038/s41598-022-24260-z (PMC9700707; doi:10.1038/s41598-022-24260-z)
Supplement: Supplementary file 1 — Supplementary Information. [file 41598_2022_24260_MOESM1_ESM.docx]

Supplementary table 1. Predicted databases used in this study with their properties.

| Resource | Version/Date | Predictions | Genes | miRNAs |
| --- | --- | --- | --- | --- |
| BCmicrO | March, 2017 | 10 682 301 | 18 418 | 580 |
| BiTargeting | April, 2017 | 5 314 760 | 18 517 | 2582 |
| CoMeTa | March, 2017 | 640 586 | 10 969 | 643 |
| Cupid | March, 2017 | 298 163 | 8411 | 1181 |
| DIANA | v5.0 | 7 112 061 | 18 529 | 1909 |
| ElMMo3 | March, 2017 | 2 837 861 | 18 179 | 997 |
| GenMir++ | March, 2017 | 5579 | 872 | 99 |
| MAMI | March, 2017 | 95 408 | 14 285 | 309 |
| MBStar | April, 2017 | 11 925 118 | 18 041 | 2031 |
| microrna.org | January, 2008 | 684 192 | 18 424 | 241 |
| MirAncesTar | March, 2017 | 36 116 591 | 18 532 | 2568 |
| mirbase | March, 2017 | 498 128 | 17 913 | 684 |
| miRcode | March, 2017 | 997 836 | 25 656 | 124 |
| mirCoX | March, 2017 | 1 716 865 | 21 749 | 79 |
| miRDB | v5.0 | 4 739 198 | 16 588 | 2571 |
| MirMAP | v.1.1 | 11 392 502 | 18 574 | 2031 |
| MirSNP | March, 2017 | 849 897 | 17 180 | 1909 |
| MirTar | March, 2017 | 686 222 | 16 556 | 1897 |
| miRTar2GO | March, 2017 | 1 164 371 | 10 890 | 366 |
| Mirza-G | April, 2016 | 4 348 927 | 16 790 | 2564 |
| MultiMiTar | March, 2017 | 429 258 | 10 986 | 473 |
| PACCMIT | February, 2012 | 363 717 | 11 735 | 1905 |
| PicTar | March, 2017 | 14 160 | 2430 | 114 |
| PITA | v6.0 | 685 848 | 18 141 | 295 |
| RepTar | March, 2017 | 2 996 265 | 17 280 | 1066 |
| RNA22 | v.2.0 | 3 127 672 | 1927 | 2584 |
| RNAhybrid | v2.1.2 | 41 306 832 | 17 448 | 2584 |
| TargetRank | March, 2017 | 342 703 | 14 241 | 525 |
| Targetscan | v7.1 | 210 146 | 11 952 | 369 |
| TargetSpy | April, 2016 | 286 654 | 15 485 | 356 |

Supplementary table 2. Effective miRNAs in atherosclerosis identified by computational databases (complete results). Number of databases that confirmed each miRNA-gene interaction was provided in each cell. Sum of the number of databases for each miRNA as P-Score was provided in the last column. As the number of databases increases, the color of each cell becomes darker.

| **miRNA** | **CETP** | **APOB** | **MTTP** | **PCSK9** | **HMGCR** | **LPA** | **APOC3** | **P-Score** |
| --- | --- | --- | --- | --- | --- | --- | --- | --- |
| hsa-miR-338-3p | 5 | 6 | 5 | 8 | 16 | 9 | 8 | 57 |
| hsa-miR-211-5p | 3 | 6 | 10 | 7 | 16 | 3 | 10 | 55 |
| hsa-miR-365a-3p | 9 | 6 | 6 | 12 | 15 | 2 | 2 | 52 |
| hsa-miR-149-5p | 4 | 11 | 5 | 13 | 13 | 2 | 3 | 51 |
| hsa-miR-204-5p | 2 | 5 | 8 | 7 | 16 | 2 | 10 | 50 |
| hsa-miR-335-5p | 2 | 2 | 12 | 12 | 16 | 2 | 2 | 48 |
| hsa-miR-139-5p | 3 | 4 | 6 | 8 | 20 | 2 | 4 | 47 |
| hsa-miR-143-3p | 5 | 4 | 10 | 12 | 9 | 2 | 5 | 47 |
| hsa-miR-548c-3p | 2 | 5 | 14 | 7 | 14 | 4 | 0 | 46 |
| hsa-miR-1237-3p | 9 | 9 | 4 | 7 | 10 | 3 | 3 | 45 |
| hsa-miR-197-3p | 5 | 3 | 11 | 10 | 8 | 3 | 4 | 44 |
| hsa-miR-21-5p | 4 | 7 | 4 | 5 | 14 | 10 | 0 | 44 |
| hsa-miR-769-5p | 4 | 9 | 8 | 10 | 12 | 0 | 0 | 43 |
| hsa-miR-152-3p | 4 | 5 | 5 | 12 | 9 | 4 | 4 | 43 |
| hsa-miR-582-5p | 4 | 3 | 11 | 6 | 15 | 2 | 2 | 43 |
| hsa-miR-145-5p | 3 | 11 | 5 | 5 | 11 | 3 | 5 | 43 |
| hsa-miR-24-3p | 3 | 10 | 3 | 17 | 6 | 0 | 3 | 42 |
| hsa-miR-224-5p | 2 | 0 | 4 | 12 | 19 | 2 | 3 | 42 |
| hsa-miR-138-5p | 4 | 6 | 3 | 14 | 5 | 7 | 3 | 42 |
| hsa-miR-221-3p | 5 | 3 | 8 | 10 | 10 | 3 | 3 | 42 |
| hsa-miR-653-5p | 2 | 3 | 16 | 4 | 15 | 0 | 2 | 42 |
| hsa-miR-223-3p | 4 | 5 | 8 | 7 | 6 | 8 | 3 | 41 |
| hsa-miR-495-3p | 2 | 3 | 14 | 3 | 12 | 5 | 2 | 41 |
| hsa-miR-16-5p | 5 | 4 | 4 | 8 | 10 | 2 | 7 | 40 |
| hsa-miR-155-5p | 9 | 6 | 8 | 3 | 14 | 0 | 0 | 40 |
| hsa-miR-222-3p | 5 | 3 | 7 | 10 | 10 | 2 | 3 | 40 |
| hsa-miR-195-5p | 4 | 4 | 4 | 8 | 10 | 3 | 7 | 40 |
| hsa-miR-9-5p | 4 | 12 | 8 | 7 | 5 | 2 | 2 | 40 |
| hsa-miR-29c-3p | 2 | 2 | 3 | 7 | 20 | 3 | 2 | 39 |
| hsa-miR-28-5p | 3 | 2 | 4 | 8 | 7 | 3 | 12 | 39 |
| hsa-miR-26b-5p | 3 | 3 | 13 | 9 | 5 | 2 | 4 | 39 |
| hsa-miR-98-5p | 4 | 4 | 13 | 8 | 6 | 0 | 4 | 39 |
| hsa-miR-590-5p | 3 | 4 | 3 | 4 | 13 | 10 | 2 | 39 |
| hsa-miR-424-5p | 4 | 4 | 4 | 9 | 11 | 2 | 4 | 38 |
| hsa-miR-20b-5p | 4 | 5 | 8 | 7 | 8 | 3 | 3 | 38 |
| hsa-miR-548p | 2 | 11 | 9 | 9 | 4 | 0 | 2 | 37 |
| hsa-miR-1270 | 4 | 4 | 5 | 11 | 4 | 0 | 9 | 37 |
| hsa-miR-1303 | 2 | 4 | 11 | 4 | 14 | 0 | 2 | 37 |
| hsa-miR-212-3p | 5 | 4 | 7 | 5 | 7 | 6 | 3 | 37 |
| hsa-miR-1207-5p | 3 | 4 | 3 | 10 | 9 | 3 | 5 | 37 |
| hsa-miR-524-5p | 2 | 3 | 10 | 9 | 11 | 0 | 2 | 37 |
| hsa-miR-577 | 2 | 3 | 9 | 9 | 12 | 0 | 2 | 37 |
| hsa-let-7g-5p | 2 | 5 | 11 | 7 | 5 | 3 | 4 | 37 |
| hsa-miR-22-3p | 4 | 5 | 4 | 7 | 8 | 4 | 5 | 37 |
| hsa-miR-626 | 2 | 4 | 4 | 4 | 11 | 9 | 3 | 37 |
| hsa-miR-27a-3p | 2 | 4 | 4 | 6 | 15 | 3 | 2 | 36 |
| hsa-miR-147a | 3 | 5 | 11 | 6 | 6 | 2 | 3 | 36 |
| hsa-miR-646 | 3 | 3 | 3 | 10 | 4 | 2 | 11 | 36 |
| hsa-miR-1321 | 8 | 3 | 5 | 10 | 5 | 2 | 3 | 36 |
| hsa-miR-520d-5p | 2 | 3 | 9 | 9 | 10 | 0 | 3 | 36 |
| hsa-miR-455-5p | 2 | 7 | 15 | 3 | 3 | 4 | 2 | 36 |
| hsa-let-7f-5p | 2 | 4 | 12 | 6 | 6 | 2 | 4 | 36 |
| hsa-miR-296-5p | 5 | 4 | 10 | 7 | 4 | 2 | 4 | 36 |
| hsa-miR-548a-3p | 2 | 2 | 8 | 4 | 14 | 2 | 4 | 36 |
| hsa-let-7b-5p | 2 | 3 | 12 | 7 | 5 | 2 | 4 | 35 |
| hsa-miR-27b-3p | 2 | 3 | 4 | 6 | 16 | 2 | 2 | 35 |
| hsa-miR-432-5p | 7 | 3 | 7 | 4 | 7 | 2 | 5 | 35 |
| hsa-miR-136-5p | 4 | 11 | 5 | 9 | 6 | 0 | 0 | 35 |
| hsa-miR-497-5p | 4 | 4 | 4 | 9 | 7 | 2 | 5 | 35 |
| hsa-miR-148a-3p | 4 | 4 | 5 | 9 | 7 | 3 | 3 | 35 |
| hsa-miR-708-5p | 3 | 2 | 3 | 8 | 5 | 3 | 11 | 35 |
| hsa-let-7c-5p | 2 | 3 | 12 | 7 | 5 | 2 | 4 | 35 |
| hsa-miR-217 | 2 | 6 | 16 | 4 | 5 | 0 | 2 | 35 |
| hsa-miR-503-5p | 3 | 4 | 4 | 9 | 5 | 2 | 8 | 35 |
| hsa-miR-511-5p | 3 | 2 | 8 | 4 | 12 | 4 | 2 | 35 |
| hsa-miR-1233-3p | 2 | 9 | 3 | 6 | 10 | 2 | 3 | 35 |
| hsa-miR-186-5p | 2 | 9 | 5 | 4 | 11 | 0 | 3 | 34 |
| hsa-miR-203a-3p | 3 | 4 | 7 | 11 | 7 | 0 | 2 | 34 |
| hsa-miR-621 | 3 | 2 | 9 | 11 | 4 | 2 | 3 | 34 |
| hsa-miR-1254 | 5 | 5 | 2 | 9 | 4 | 2 | 7 | 34 |
| hsa-miR-182-5p | 3 | 8 | 3 | 7 | 6 | 5 | 2 | 34 |
| hsa-miR-129-5p | 2 | 4 | 13 | 6 | 6 | 0 | 3 | 34 |
| hsa-miR-218-5p | 2 | 11 | 7 | 4 | 6 | 2 | 2 | 34 |
| hsa-miR-578 | 0 | 2 | 7 | 3 | 13 | 0 | 9 | 34 |
| hsa-miR-216b-5p | 5 | 3 | 6 | 3 | 12 | 3 | 2 | 34 |
| hsa-miR-150-5p | 5 | 6 | 5 | 7 | 5 | 3 | 3 | 34 |
| hsa-miR-1275 | 5 | 4 | 5 | 6 | 7 | 2 | 5 | 34 |
| hsa-miR-760 | 3 | 3 | 4 | 5 | 14 | 0 | 5 | 34 |
| hsa-miR-31-5p | 2 | 5 | 5 | 8 | 8 | 2 | 3 | 33 |
| hsa-miR-92a-3p | 2 | 5 | 4 | 5 | 12 | 2 | 3 | 33 |
| hsa-miR-1297 | 2 | 4 | 11 | 9 | 4 | 0 | 3 | 33 |
| hsa-miR-544a | 5 | 4 | 6 | 8 | 5 | 2 | 3 | 33 |
| hsa-miR-1283 | 3 | 3 | 4 | 8 | 13 | 0 | 2 | 33 |
| hsa-miR-940 | 3 | 5 | 3 | 7 | 4 | 2 | 9 | 33 |
| hsa-let-7d-5p | 2 | 3 | 11 | 7 | 6 | 0 | 4 | 33 |
| hsa-miR-922 | 3 | 3 | 11 | 7 | 6 | 0 | 3 | 33 |
| hsa-miR-526b-5p | 3 | 4 | 8 | 4 | 10 | 2 | 2 | 33 |
| hsa-miR-105-5p | 2 | 4 | 9 | 4 | 7 | 5 | 2 | 33 |
| hsa-miR-548c-5p | 2 | 6 | 5 | 5 | 8 | 5 | 2 | 33 |
| hsa-miR-620 | 4 | 4 | 5 | 10 | 4 | 0 | 6 | 33 |
| hsa-miR-532-5p | 3 | 3 | 4 | 12 | 4 | 2 | 5 | 33 |
| hsa-miR-15b-5p | 3 | 3 | 4 | 7 | 9 | 0 | 6 | 32 |
| hsa-miR-939-5p | 5 | 2 | 5 | 10 | 4 | 2 | 4 | 32 |
| hsa-miR-615-3p | 3 | 12 | 3 | 7 | 4 | 0 | 3 | 32 |
| hsa-miR-661 | 4 | 4 | 3 | 10 | 4 | 0 | 7 | 32 |
| hsa-miR-214-3p | 4 | 3 | 6 | 9 | 7 | 0 | 3 | 32 |
| hsa-miR-34c-5p | 3 | 7 | 4 | 8 | 3 | 3 | 4 | 32 |
| hsa-miR-106a-5p | 3 | 3 | 7 | 7 | 7 | 3 | 2 | 32 |
| hsa-miR-17-5p | 3 | 4 | 7 | 7 | 7 | 2 | 2 | 32 |
| hsa-miR-599 | 3 | 3 | 14 | 3 | 5 | 2 | 2 | 32 |
| hsa-miR-3646 | 0 | 6 | 9 | 5 | 10 | 0 | 2 | 32 |
| hsa-miR-655-3p | 2 | 3 | 4 | 2 | 12 | 7 | 2 | 32 |
| hsa-miR-548d-5p | 2 | 6 | 5 | 4 | 9 | 4 | 2 | 32 |
| hsa-miR-1202 | 5 | 9 | 3 | 7 | 3 | 2 | 3 | 32 |
| hsa-miR-92b-3p | 2 | 4 | 3 | 5 | 12 | 2 | 3 | 31 |
| hsa-miR-7-5p | 4 | 3 | 5 | 8 | 6 | 2 | 3 | 31 |
| hsa-miR-449b-5p | 3 | 8 | 4 | 8 | 3 | 2 | 3 | 31 |
| hsa-miR-498 | 3 | 2 | 6 | 8 | 7 | 2 | 3 | 31 |
| hsa-miR-373-3p | 3 | 2 | 3 | 7 | 7 | 6 | 3 | 31 |
| hsa-miR-3662 | 0 | 4 | 9 | 6 | 8 | 2 | 2 | 31 |
| hsa-miR-519d-3p | 2 | 7 | 7 | 4 | 6 | 3 | 2 | 31 |
| hsa-miR-548a-5p | 2 | 6 | 5 | 4 | 8 | 4 | 2 | 31 |
| hsa-miR-1915-3p | 3 | 4 | 3 | 12 | 5 | 0 | 3 | 30 |
| hsa-miR-4715-5p | 3 | 3 | 8 | 8 | 8 | 0 | 0 | 30 |
| hsa-miR-302c-3p | 3 | 3 | 6 | 6 | 6 | 4 | 2 | 30 |
| hsa-miR-548i | 2 | 5 | 5 | 4 | 8 | 4 | 2 | 30 |
| hsa-miR-520c-3p | 3 | 4 | 5 | 6 | 5 | 4 | 3 | 30 |
| hsa-miR-920 | 4 | 3 | 4 | 9 | 3 | 2 | 5 | 30 |
| hsa-miR-671-5p | 3 | 4 | 3 | 8 | 5 | 2 | 5 | 30 |
| hsa-miR-3065-3p | 2 | 3 | 2 | 10 | 12 | 0 | 0 | 29 |
| hsa-miR-26a-5p | 2 | 2 | 10 | 8 | 4 | 0 | 3 | 29 |
| hsa-miR-3619-5p | 3 | 3 | 6 | 8 | 6 | 0 | 3 | 29 |
| hsa-miR-302d-3p | 3 | 3 | 4 | 6 | 6 | 5 | 2 | 29 |
| hsa-miR-548g-3p | 2 | 2 | 7 | 2 | 12 | 2 | 2 | 29 |
| hsa-miR-1909-3p | 3 | 3 | 4 | 7 | 7 | 0 | 5 | 29 |
| hsa-miR-193b-3p | 3 | 3 | 3 | 3 | 10 | 3 | 3 | 28 |
| hsa-miR-32-5p | 0 | 2 | 4 | 5 | 12 | 3 | 2 | 28 |
| hsa-miR-149-3p | 5 | 3 | 3 | 10 | 4 | 0 | 3 | 28 |
| hsa-miR-449c-5p | 3 | 9 | 0 | 7 | 3 | 0 | 6 | 28 |
| hsa-miR-135a-5p | 3 | 5 | 5 | 7 | 3 | 3 | 2 | 28 |
| hsa-miR-302a-3p | 3 | 3 | 4 | 6 | 6 | 4 | 2 | 28 |
| hsa-miR-216a-5p | 5 | 3 | 6 | 3 | 6 | 3 | 2 | 28 |
| hsa-miR-4251 | 0 | 2 | 5 | 4 | 10 | 0 | 7 | 28 |
| hsa-miR-34b-5p | 2 | 8 | 3 | 7 | 3 | 0 | 5 | 28 |
| hsa-miR-191-5p | 2 | 3 | 3 | 11 | 5 | 0 | 3 | 27 |
| hsa-miR-29b-3p | 0 | 0 | 2 | 6 | 19 | 0 | 0 | 27 |
| hsa-miR-302b-3p | 3 | 3 | 3 | 5 | 6 | 5 | 2 | 27 |
| hsa-miR-584-5p | 2 | 11 | 3 | 4 | 5 | 0 | 2 | 27 |
| hsa-miR-302b-5p | 2 | 9 | 5 | 2 | 9 | 0 | 0 | 27 |
| hsa-miR-30c-1-3p | 3 | 3 | 4 | 8 | 4 | 0 | 5 | 27 |
| hsa-miR-93-5p | 2 | 4 | 6 | 6 | 6 | 2 | 0 | 26 |
| hsa-miR-4635 | 0 | 4 | 8 | 4 | 9 | 0 | 0 | 25 |
| hsa-miR-454-3p | 2 | 3 | 4 | 5 | 6 | 3 | 2 | 25 |
| hsa-miR-429 | 0 | 3 | 5 | 0 | 6 | 0 | 10 | 24 |
| hsa-miR-4263 | 0 | 2 | 4 | 3 | 10 | 5 | 0 | 24 |
| hsa-miR-187-5p | 5 | 0 | 3 | 10 | 5 | 0 | 0 | 23 |
| hsa-miR-30e-3p | 0 | 2 | 6 | 4 | 8 | 3 | 0 | 23 |
| hsa-miR-124-3p | 0 | 3 | 2 | 10 | 4 | 1 | 2 | 22 |
| hsa-miR-15a-5p | 0 | 2 | 3 | 6 | 7 | 0 | 4 | 22 |
| hsa-miR-192-5p | 3 | 3 | 3 | 6 | 5 | 0 | 2 | 22 |
| hsa-miR-205-5p | 0 | 4 | 5 | 6 | 7 | 0 | 0 | 22 |
| hsa-miR-3678-3p | 0 | 0 | 9 | 4 | 5 | 4 | 0 | 22 |
| hsa-miR-551b-5p | 0 | 0 | 9 | 0 | 7 | 5 | 0 | 21 |
| hsa-miR-4711-3p | 0 | 0 | 9 | 4 | 3 | 5 | 0 | 21 |
| hsa-miR-380-3p | 0 | 0 | 9 | 0 | 11 | 0 | 0 | 20 |
| hsa-miR-5688 | 0 | 0 | 8 | 2 | 7 | 3 | 0 | 20 |
| hsa-miR-30c-5p | 2 | 3 | 3 | 2 | 4 | 2 | 2 | 18 |
| hsa-miR-372-3p | 0 | 0 | 2 | 5 | 5 | 4 | 2 | 18 |
| hsa-miR-489-3p | 0 | 4 | 2 | 3 | 4 | 5 | 0 | 18 |
| hsa-miR-30e-5p | 0 | 2 | 11 | 0 | 4 | 0 | 0 | 17 |
| hsa-miR-4503 | 0 | 0 | 2 | 0 | 8 | 0 | 6 | 16 |
| hsa-miR-423-5p | 0 | 0 | 2 | 7 | 0 | 3 | 3 | 15 |
| hsa-miR-3529-3p | 0 | 0 | 6 | 0 | 7 | 0 | 0 | 13 |
| hsa-miR-500b-3p | 0 | 0 | 2 | 4 | 2 | 0 | 0 | 8 |

Supplementary table 3. Binding characteristics of miRNAs with target genes.

| **miRNA** | **Gene** | **Confidence scores** | **Predicted free energy**  **(kCal/mol)** | **Position of target site** |
| --- | --- | --- | --- | --- |
| miR-124 | HMGCR | 0.24919742 | -25.4 | 36 |
|  | PCSK9 | 0.27949999 | -31.9 | 227 |
|  | CETP | 0.163443479067538 | -35.1 | 104 |
|  | MTTP | 0.215091232269613 | -24.6 | 2179 |
|  | APOB | 0.622066749489982 | -28.9 | 9986 |
|  | LPA | 0.117251415260855 | -24.5 | 5995 |
|  | APOC3 | 0.0481657328031938 | -24.8 | 316 |
| miR-16 | HMGCR | 0.657893519028436 | -27.2 | 665 |
|  | PCSK9 | 0.24919742 | -26.2 | 1255 |
|  | CETP | 0.657893519028436 | -23.3 | 238 |
|  | MTTP | 0.622066749489982 | -23.6 | 3434 |
|  | APOB | 0.622066749489982 | -26.3 | 135 |
|  | LPA | 0.0481657328031938 | -22.1 | 5281 |
|  | APOC3 | 0.0829781056820632 | -20.7 | 172 |

Supplementary table 4. Prediction of interaction between miRNAs and their target genes using RNAhybrid 2.2.

| **miRNA** | **Gene** | Prediction interaction |
| --- | --- | --- |
| miR-124 | HMGCR | 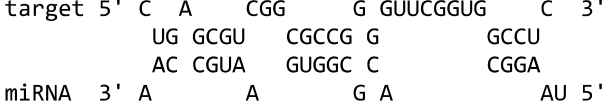 |
|  | PCSK9 | 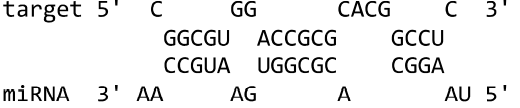 |
|  | CETP | 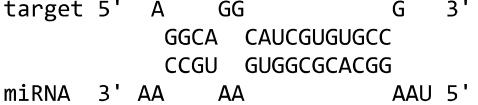 |
|  | MTTP | 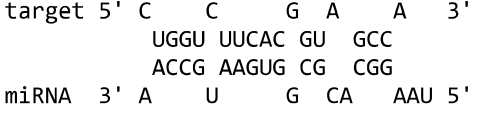 |
|  | APOB | 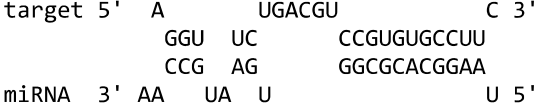 |
|  | LPA | 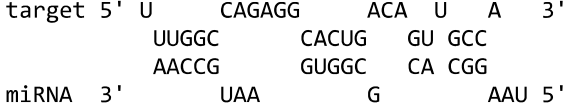 |
|  | APOC3 | 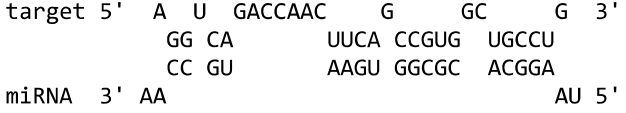 |
| miR-16 | HMGCR | 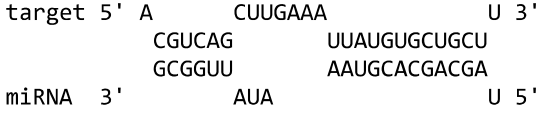 |
|  | PCSK9 | 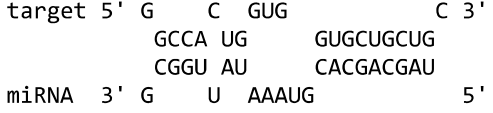 |
|  | CETP | 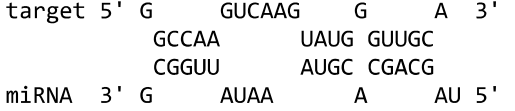 |
|  | MTTP | 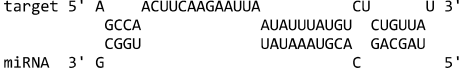 |
|  | APOB | 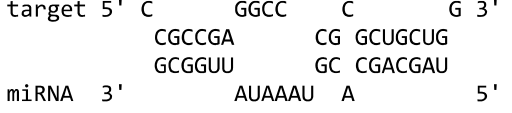 |
|  | LPA | 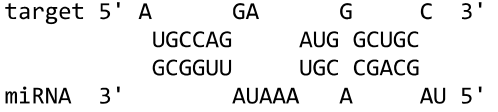 |
|  | APOC3 | 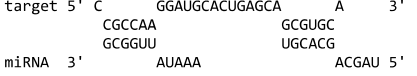 |
